# Aqueous and Organic Solvent-Extracts of Selected South African Medicinal Plants Possess Antimicrobial Activity against Drug-Resistant Strains of *Helicobacter pylori*: Inhibitory and Bactericidal Potential

**DOI:** 10.3390/ijms12095652

**Published:** 2011-09-02

**Authors:** Collise Njume, Afolayan A. Jide, Roland N. Ndip

**Affiliations:** 1 School of Biological and Environmental Sciences, Faculty of Science and Agriculture, University of Fort Hare, P/Bag X1314, Alice 5700, South Africa; E-Mails: njumecol@yahoo.com (C.N.); aafolayan@ufh.ac.za (A.A.J.); 2 Department of Biochemistry and Microbiology, Faculty of Science, University of Buea, Box 63, Buea 00237, Cameroon

**Keywords:** drug discovery, *Helicobacter pylori*, medicinal plants, antimicrobial activity

## Abstract

The aim of this study was to identify sources of cheap starting materials for the synthesis of new drugs against *Helicobacter pylori*. Solvent-extracts of selected medicinal plants; *Combretum molle*, *Sclerocarya birrea*, *Garcinia kola*, *Alepidea amatymbica* and a single *Strychnos* species were investigated against 30 clinical strains of *H. pylori* alongside a reference control strain (NCTC 11638) using standard microbiological techniques. Metronidazole and amoxicillin were included in these experiments as positive control antibiotics. All the plants demonstrated anti-*H. pylori* activity with zone diameters of inhibition between 0 and 38 mm and 50% minimum inhibitory concentration (MIC_50_) values ranging from 0.06 to 5.0 mg/mL. MIC_50_ values for amoxicillin and metronidazole ranged from 0.001 to 0.63 mg/mL and 0.004 to 5.0 mg/mL respectively. The acetone extracts of *C. molle* and *S. birrea* exhibited a remarkable bactericidal activity against *H. pylori* killing more than 50% of the strains within 18 h at 4× MIC and complete elimination of the organisms within 24 h. Their antimicrobial activity was comparable to the control antibiotics. However, the activity of the ethanol extract of *G. kola* was lower than amoxicillin (*P* < 0.05) as opposed to metronidazole (*P* > 0.05). These results demonstrate that *S. birrea*, *C. molle* and *G. kola* may represent good sources of compounds with anti-*H. pylori* activity.

## 1. Introduction

*Helicobacter pylori*, a gram negative microearophilic helical bacillus inhabits the stomach of approximately half of the human population, in whom it may persist for a life time, making it one of the most successful human pathogens [1]. Infection with this organism is strongly associated with chronic active gastritis, peptic ulcer and mucosa associated lymphoid tissue (MALT) lymphoma [2]. The organism is classified by the World Health Organization and the International agency for Research on Cancer as a class 1 carcinogen [3].

Eradication of the organism from the stomach results in significant remission from the above diseases [4]. Current eradication regimens involve the use of combination therapies; a proton pump inhibitor (PPI) or bismuth compounds and two antibiotics, most commonly clarithromycin and metronidazole or amoxicillin [5], with an expected success rate of 80%–90% [6]. However, *H. pylori* infection is still difficult to treat as eradication failure rate remains at 10%–40% [7,8].

Antimicrobial resistance to current regimens is increasingly recognized as a major cause of treatment failure [9]. With primary susceptibility patterns becoming less predictable, it is not uncommon to find other stronger antibiotics, particularly from the flouroquinolone group, being part of the treatment regimen [10], but *H. pylori* is developing resistance to these drugs too [11]. Other problems, including undesirable side effects (nausea, vomiting, diarrhea, stomach ache) and poor patient compliance are associated with significant levels of treatment failure and contraindications for some patients [8]. In addition, combination therapy is not readily affordable and some of the drugs are not available in some rural settings in the developing world. These factors and others have necessitated the search for alternative treatment regimens with highly selective activity against the organism, without the risk of resistance or other untoward effects.

The use of medicinal plants for the treatment of diseases is a common phenomenon in most African countries, particularly in areas where medical health facilities are not readily accessible or affordable [12]. Thus, plants would seem to be a logical source of new anti-*H. pylori* compounds. In fact, our previous studies have documented that some medicinal plant extracts have antibacterial activity against *H. pylori* [13–15].

*Combretum molle*, *Sclerocarya birrea*, *Garcinia kola*, *Alepidea amatymbica* and some *Strychnos* species have found ethno medicinal uses in the treatment of gastritis, peptic ulcer and other *H. pylori*-associated morbidities in South Africa and other African countries [6,16,17]. The medicinal properties of most of these plants have been a subject of numerous investigations [18–20]. However, reports on their anti-*H. pylori* activity are lacking. This is surprising, given that *H. pylori* antimicrobial resistance to current treatment regimens is increasingly recognized as a major cause of treatment failure. This study was therefore carried out to evaluate the antimicrobial activity of the selected medicinal plants on clinical isolates of *H. pylori* as part of an ongoing effort to identify potential sources of cheap starting materials for the synthesis of new drugs against this carcinogenic organism.

## 2. Results and Discussion

### 2.1. Extract Yield

**Figure 1:**
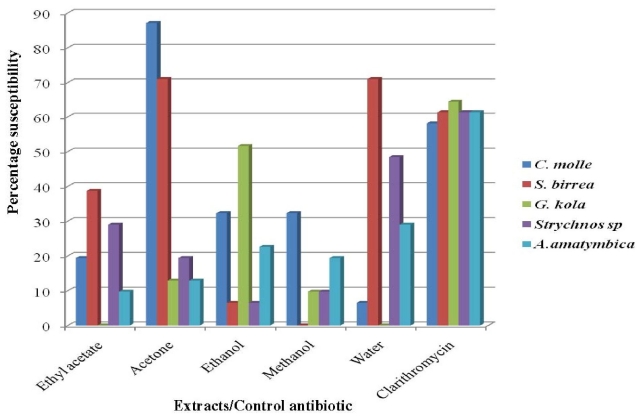
Activity of plant crude extracts on *H. pylori* by agar well diffusion method. Percentage susceptibilities are representative of the number of strains whose zone of inhibition diameter is ≥14 mm.

The total amount of crude extract obtained with the different solvents shows that methanol was quantitatively the best solvent for extraction in all the plants while ethyl acetate had the lowest yields in four of the five plants studied (Table 1). The efficiency of methanol in the extraction of phytochemicals has been reported in other studies [13,21,22]. Our results seem to be consistent with others confirming methanol as a good solvent for extraction of bioactive compounds from plants as it gave the highest yield in all five plants studied. However, the extract yield may not always relate proportionally with its activity as revealed by the activity of the methanol extract herein (Figure 1). Nevertheless, the activity demonstrated by the methanol extracts gives an indication of their potential as useful bioactive substances.

### 2.2. Antimicrobial Activity of Extracts

All the plant crude extracts tested demonstrated anti-*H. pylori* activity with zone diameters of inhibition between 0 and 38 mm. The highest mean zone diameter of 17.4 ± 5.0 mm was recorded for the acetone extract of *C. molle* (Table 2) which also gave the highest percentage susceptibility of 87.1%, followed by acetone and aqueous extracts of *S. birrea* with 71% each (Figure 1). *Strychnos* species and *A. amatymbica* gave percentage susceptibilities of less than 50%.

MIC values of the plant extracts ranged from 0.06 to 5.0 mg/mL and those of amoxicillin and metronidazole ranged from 0.001 to 0.63 mg/mL and 0.004 to 5.0 mg/mL respectively (Table 3). Of all the extracts analyzed, the acetone extracts of *S. birrea* and *C. molle* exhibited interesting activities with MIC values ranging from 0.06 to 2.50 mg/mL and 0.08 to 2.50 mg/mL respectively with no significant differences between their activity and that of amoxicillin (*P* > 0.05); as opposed to metronidazole (*P* < 0.05). However, the activity of the ethanol extract of *G. kola* was significantly different from amoxicillin (*P* < 0.05) as opposed to metronidazole (*P* > 0.05).

The antimicrobial activity of the acetone extracts of *C. molle* and *S. birrea* (Figure 1) may imply that acetone could be a better solvent for the extraction of anti-*H. pylori* compounds from both plants. Eloff [23] in a comparison of acetone, ethanol, methanol, methylenedichloride, methanol/chloroform/water and water observed acetone to be the best in terms of the diversity of compounds extracted. Such diverse compounds may act in synergy and produce a greater antimicrobial effect on *H. pylori*, thus resulting in high susceptibility patterns and low MIC values observed (Table 3).

The acetone extracts of *C. molle* and *S. birrea* seem to possess significant inhibitory activity against *H. pylori* when compared with metronidazole and amoxicillin. Crude extracts of other plants have been shown to have comparable activity with amoxicillin and better activity when compared with metronidazole against *H. pylori* in other studies [1,24]. This is quite remarkable considering that the antibiotics are in the purified and concentrated form whereas the extracts are crude and may harbor both pharmacologically and non-pharmacologically active compounds with the chance of some compounds having a masking effect over others. This is an indication that the acetone extracts of *C. molle* and *S. birrea* may contain therapeutically useful compounds against *H. pylori* infections.

The results of this study indicate a 96.7% resistance of the strains tested to metronidazole and amoxicillin (Table 3). This high resistance is probably due to the fact that all the clinical strains used were isolated from patients with a history of recurrent peptic ulcer infection in the hospital, where there is a high chance of finding resistant bacteria. *H. pylori*-resistance to metronidazole has been an existing problem [1,25], especially in the developing world and can be attributed to wide usage of this drug in the treatment of other infections, especially parasitic and gynaecological infections [26]. Our results are consistent with other studies indicating that amoxicillin resistance, previously reported to be rare, is rising at an alarming rate, especially in third world countries where drug prescription practices are probably not adhered to [1,27]. The consequence will be an increase in eradication failure, encouraging clinicians to resort to second and third line therapies, some of which may require drug combinations that are not readily available in South Africa. Therefore, the present results indicate the need to test the antimicrobial susceptibility of *H. pylori* in the country to guide empiric treatment.

A careful look at Table 3 shows that patients may be co-infected with mixed antibiotic-resistant/ susceptible strains at the same time, in the same or different sites of the stomach which is in concordance with the findings of many other researchers [28–30]. It therefore becomes imperative for gastric biopsies to be collected from different parts of the stomach and to analyze the susceptibility patterns of bacteria isolated from different sites (antrum/corpus) of the same patient.

### 2.3. Bactericidal Activity

**Figure 2:**
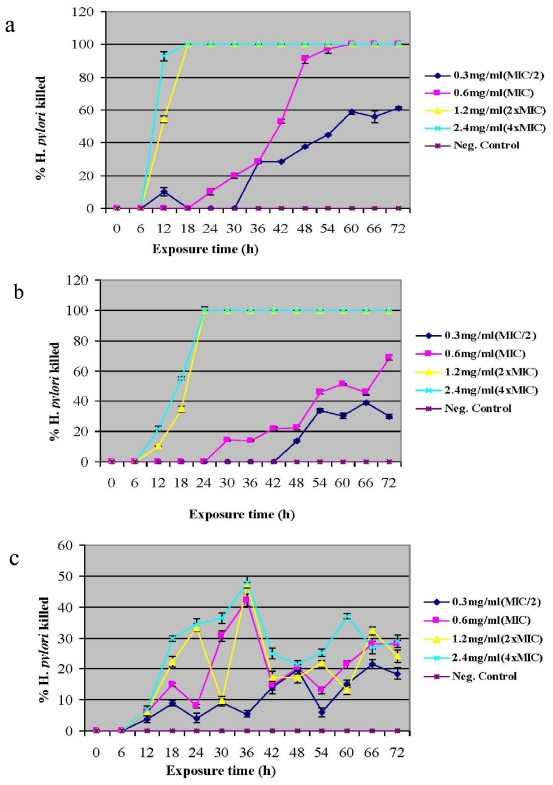
(**a**) Rate of killing of *H. pylori* by the acetone extract of *S. birrea*; (**b**) Rate of killing of *H. pylori* by the acetone extract of *C. molle*; (**c**) Rate of killing of *H. pylori* by the aqueous extract of *S. birrea*. Percentage of *H. pylori* killed represents mean values of triplicate determinations for the strains PE2A, PE14C and PE93A. Error bars represent the standard deviation (SD). Very small error bars have been obscured by data symbols. Neg. Control, extract-free cells.

There seem to be a linear relationship exhibited by the bactericidal activity of the extracts with time and concentration. For example, at 1.2 mg/mL (2× MIC) a killing rate of 54.4% of *H. pylori* was recorded for the acetone extract of *S. birrea* after 12 h and complete eradication of the organisms after 18 h. When this concentration was doubled (2.4 mg/mL) (4× MIC), the killing rate was more than 90% after 12 h with complete eradication of the organisms after 18 h (Figure 2a). The relationship was almost the same for the acetone extract of *C. molle* with a 35.0% and 55.0% killing within 18 h at 1.2 mg/mL (2× MIC) and 2.4 mg/mL (4× MIC) respectively; and complete elimination of the organisms within 24 h at the same concentrations (Figure 2b). These findings seem to corroborate the observations of Akinpelu *et al.* [31] who reported the rate of killing of *Escherichia coli* and *Bacillus subtilis* by the aqueous fractions of *Afzelia africana* to be dependent on time and extract concentration. However *H. pylori* was not among the organisms tested and unlike in Akinpelu’s study where fractions were used, our extracts are still crude with better activity expected upon fractionation and purification from pharmacologically inactive substances that may interfere with the assay.

A relatively lower bactericidal activity was however noticed with the aqueous extract of *S. birrea*. Even though there was a consistent increase in the percentage of *H. pylori* killed with time at MIC, 2× MIC and 4× MIC, this was not maintained to the end of the assay (Figure 2c). The peak bactericidal activity exhibited by the extract at the 36th hour coincides with the exponential growth phase of the organisms, a time during which they are most sensitive to antimicrobial agents. It is likely that some of the organisms that survived this period were already becoming resistant to the extract after 36 h. From all indications, the aqueous extract of *S. birrea* was only weakly bactericidal.

It is worth mentioning that the literature is rich with information on the very limited activity or complete non responsiveness of microorganisms to plant aqueous extracts [19,32,33]. Our study lays credence to this as aqueous extracts of 4 of the 5 plants studied recorded percentage susceptibilities of less than 50%. However, these results also provide evidence that water could still be a good solvent in the extraction of anti-*H. pylori* compounds from *S. birrea* as it gave an activity of 71% in the screening (Figure 1). This is particularly important in the study area and other rural settings in Africa where organic solvents reported to be better solvents for extraction [13,34] may not be within the reach of the traditional healers, as most of them make use of aqueous extracts in their practice. Equally important is the fact that the use of water is environmentally safer than most organic solvents.

Crude ethanol extracts of *G. kola* seeds exhibited a better activity when compared to metronidazole (*P* > 0.05), a pure compound. This is not surprising given the high *H. pylori* resistance to metronidazole in South Africa [25]. Equally important is the fact that the seeds of this plant are reported to be rich in flavonoids and tannins, substances with great antimicrobial potentials and health-promoting abilities [35,36]. Synergistic interactions between some of these compounds may produce greater antimicrobial activity against *H. pylori*. The antimicrobial activity demonstrated by the ethanolic extract of *G. kola* is an indication of its potential as a possible source of new compounds against *H. pylori* infections.

The antimicrobial activity demonstrated by crude extracts of the lone *Strychnos* species and *A. amatymbica* was relatively low (Figure 1). It is likely that the amount of active ingredients in these plant extracts may not occur in quantities large enough to produce significant activity. Such extracts upon fractionation, purification and concentration of the active ingredients may still lead to the isolation of therapeutically useful compounds against *H. pylori* or other infectious organisms.

## 3. Experimental

### 3.1. Bacterial Strains

A total of thirty strains of *H. pylori* were isolated from gastric biopsies of patients with recurrent peptic ulcer infection undergoing endoscopy at the Livingstone hospital, Port Elizabeth and confirmed following our previously reported scheme [1]. A reference strain of *H. pylori* (NCTC 11638) was also included in the study. Ethical clearance was obtained from the Eastern Cape Department of Health and the Govan Mbeki Research and Development Centre, University of Fort Hare. Specimens were only collected from patients who had given consent and had not received antibiotics or PPIs for at least a week. Pure cultures were suspended in eppendorf tubes containing 1 mL of Brain Heart Infusion (BHI) broth (Oxoid LTD, Basingstoke, Hampshire, England) and 20% glycerol and stored at −80 °C (Ilshin^®^, Model DF 9007, Sanyo, Osaka, Japan) for future experiments.

### 3.2. Preparation of Plant Material

The plants were selected based on ethno botanical information. The stem barks of *Combretum molle* R. Br. Ex G. Don (Combretaceae) and *Sclerocarya birrea* A. Rich Hochst (Anacardiaceae) were harvested in the vicinity of the University of Venda, Limpopo Province. The stem bark of a single *Strychnos* species Gilg. (Loganiaceae) and the roots/rhizomes of *Alepidea Amatymbica* Eckl. and Zeyh (Apiaceae) were purchased from the traditional medicine market in King William’s town and amayeza yesiXhosa (Xhosa medicine) stores in Alice. The seeds of *G. kola* Heckel (Guttiferae) were purchased from a local market in Cameroon. *C. molle* was identified by botanists at the University of Venda where vouchers have been deposited. The rest of the plants were identified by botanists in the phytomedicine research unit at the University of Fort Hare and vouchers deposited in the Giffen herbarium. The plant materials were washed, separately chopped into small pieces and dried in a hot air oven (Schwabach, Germany) at 40 °C for 48 h. The plant material was powdered (ATO mix, Torrington, CT, USA) and stored in air tight containers in a dark cupboard for future use.

### 3.3. Preparation of Plant Extract

Exactly 300 g of dried powdered plant material was macerated separately in 600 mL of concentrated ethyl acetate, acetone, ethanol and methanol (Merck, Wadeville, Gauteng, South Africa) in large glass bottles (SIMAX, Sazava, Czech Republic). Aqueous extracts were also prepared by soaking same amount of plant material in tap water. The bottles were labeled and put in an orbital shaker (New Brunswick Scientific, Edison, NJ, USA) for 48 h. The plant extracts were centrifuged at 1006.2 g for 5 min, and filtered using a fritted filter funnel of pore size 60 Å. The procedure was repeated twice and the three extracts combined and evaporated to dryness under vacuum in a rotary evaporator (BUCHI rota vapour, Flavil/Schweiz, Switzerland) set at temperatures depending on the solvent in use. The filtrate obtained from aqueous extracts was lyophilized [24]. The dried crude extracts were collected in clean universal bottles and left open in a bio-safety class 11 cabinet (Vivid Air, Durban, South Africa) for complete evaporation of residual solvents. A 2-g sample of each extract was used for the preliminary bioassay, and where possible, another 2 g was kept in the extract bank. Stock solutions were prepared by dissolving the extracts in dimethyl sulphoxide (DMSO) or acetone; 10% and 80% respectively (neither DMSO nor acetone were inhibitory to the *H. pylori* strains at the tested concentrations).

### 3.4. Screening of Crude Extracts for Anti-H. pylori Activity

This was achieved by the agar well diffusion method as previously reported [37]. Briefly, *H. pylori* inocula prepared at McFarland’s turbidity standard 2 was plated onto BHI agar supplemented with 5% horse blood and Skirrow’s supplement (Oxoid, England). The inocula was evenly spread on the plate and allowed to dry for 15 min. Wells (6 mm in diameter) were punched into the agar using a sterile stainless steel borer and filled with 65 μL of the extract at 100 mg/mL. Sixty five micro liters of 0.05 μg/mL clarithromycin and 10% DMSO were included in all experiments as positive and negative controls respectively. The plates were incubated under microaerophilic conditions (Anaerocult, Oxoid, UK) at 37 °C for 72 h after which the diameters of zones of inhibition were measured in millimeters. The experiment was repeated once and mean zones were recorded. A *Helicobacter pylori* control strain, NCTC 11638-inoculated plate was included in all the experiments.

### 3.5. Determination of 50% Minimum Inhibitory Concentration (MIC_50_)

Active extracts that had given a percentage susceptibility of ≥50% by agar well diffusion were selected for further determination of MICs by the micro broth dilution method [38], performed in 96-well plates. The test extracts were prepared at a concentration of 5.0 mg/mL and filtered through a 2.0 μm filter (Acrodisc Pall, MI, USA). Two-fold dilutions of each extract were made in the test wells in BHI broth supplemented with 5% horse serum and Skirrow’s supplement (Oxoid, England). The final extract concentrations ranged from 0.002 to 5.0 mg/mL. Twenty micro liters of an 18-h old broth culture of *H. pylori* (McFarland’s turbidity standard 2) suspension was added to 180 μL of extract-containing culture medium. Control wells were prepared with culture medium only and bacterial suspension and broth only respectively. Metronidazole and amoxicillin were run alongside each batch of extracts at 0.002–5.0 mg/mL and 0.0005–5.0 mg/mL concentration ranges respectively. An automatic ELISA micro plate reader (Tokyo, Japan) adjusted to 620 nm was used to measure the absorbance of the plates before and after 72-h incubation at 37 °C under microaerophilic conditions. The absorbencies were compared to detect an increase or decrease in bacterial growth and the values plotted against concentration. The lowest concentration of the test extract resulting in inhibition of 50% of bacterial growth was recorded as the MIC.

### 3.6. Determination of Rate of Killing

The most active plant crude extracts with mean MIC values ≤1.0 mg/mL were considered for the rate of kill experiments. The rate and extend of killing of *H. pylori* by the acetone extracts of *C. molle* and *S. birrea* as well as the aqueous extract of the latter were determined as described by Akinpelu *et al.* [31] with slight modifications. The turbidity of an 18-h old broth culture of *H. pylori* was standardized to 10^8^ CFU/mL. One mill of this suspension was added to 9 mL of BHI broth supplemented with 5% horse serum and Skirrow’s reagents and containing the extract at 1/2× MIC, MIC, 2× MIC and 4× MIC in McCartney bottles (Oxoid, England). A negative control bottle was prepared with bacterial suspension and broth only.

A 0.1-mL sample was plated from these bottles before incubation at 37 °C under microaerophilic conditions. Exactly 0.5 mL volume of each suspension was withdrawn at 6-h interval for 72 h and transferred to 4.5 mL of BHI broth recovery medium containing 3% “Tween 80” to neutralize the effects of the antimicrobial extract carry-overs from the test organisms. The suspension was 10-fold serially diluted in sterile saline (0.9% w/v sodium chloride) and plated in triplicates. The plates were incubated at 37 °C for 72 h under microaerophilic conditions and viable counts determined. Counts obtained from extract containing culture bottles were compared with those of the negative control bottle and the percentage of bacteria killed was determined.

### 3.7. Statistical Analysis

The statistical packages used for analysis were Excel (Microsoft, Redmond, WA, USA) and SPSS version 17.0 (2009, SPSS, Inc., Chicago, IL, USA). One-way analysis of variance (ANOVA) was used to compare the mean difference in inhibitory activities of extracts and antibiotics, followed by Turkey’s *post-hoc* test. Differences were considered significant at *P* < 0.05.

## 4. Conclusion

All the plants studied herein demonstrated considerable anti-*H. pylori* activity. This study may thus serve as preliminary scientific validation of their folkloric uses in the treatment of infections due to *H. pylori* in South Africa. *C. molle*, *S. birrea* and *G. kola* may contain compounds mostly in the acetone, acetone/aqueous and ethanol crude extracts respectively that could be used as lead molecules for the synthesis of novel drugs against this carcinogenic organism. Isolation and characterization of the plant active components is our next line study.

## Figures and Tables

**Table 1:** Crude extract yield.

Percentage of plant crude extract obtained with different solvents						
Plant	Family	EA	A	E	M	W
*Combretum molle* R. Br. Ex G. Don	Combretaceae	0.4	1.5	1.1	1.7	1.0
*Sclerocarya birrea* A. Rich (Hochst)	Anacardiaceae	0.9	3.3	4.2	5.2	1.9
*Garcinia kola* Heckel	Guttiferae	0.5	1.5	1.5	3.2	1.8
*Strychnos* species Gilg.	Loganiaceae	0.4	0.6	0.7	4.7	2.3
*Alepidea amatymbica* Eckl. and Zeyh	Apiaceae	4.6	1.9	1.2	5.6	2.9

EA, Ethyl acetate; A, Acetone; E, Ethanol; M, Methanol; W, Water.

**Table 2:** Screening of plant crude extracts for anti-*H. pylori* activity.

Plant	Solvent extract	Mean zone diameter ± SD (mm)	Inhibition diameter range (mm)
*C. molle*	Ethyl acetate	10.7 ± 4.7	0–21
	Acetone	17.4 ± 5.0	10–38
	Ethanol	12.9 ± 4.7	7–35
	Methanol	13.1 ± 5.3	7–32
	Water	2.7 ± 5.5	0–20
	Positive control	13.5 ± 8.7	0–32

*S. birrea*	Ethyl acetate	13.2 ± 2.8	8–20
	Acetone	14.7 ± 2.5	11–21
	Ethanol	3.3 ± 5.0	0–16
	Methanol	3.0 ± 4.4	0–11
	Water	15.0 ± 2.7	10–20
	Positive control	16.6 ± 7.4	0–32

*G. kola*	Ethyl acetate	5.1 ± 4.6	0–13
	Acetone	8.8 ± 5.2	0–25
	Ethanol	9.2 ± 7.2	0–19
	Methanol	7.1 ± 5.8	0–20
	Water	1.0 ± 2.6	0–8
	Positive control	16.1 ± 8.3	0–32

*Strychnos* sp.	Ethyl acetate	10.1 ± 6.4	0–26
	Acetone	8.8 ± 6.8	0–26
	Ethanol	4.9 ± 6.2	0–18
	Methanol	5.5 ± 5.9	0–15
	Water	11.9 ± 5.6	0–23
	Positive control	15.5 ± 7.3	0–32

*A. amatymbica*	Ethyl acetate	8.5 ± 4.8	0–17
	Acetone	7.0 ± 6.5	0–20
	Ethanol	6.7 ± 6.7	0–20
	Methanol	6.1 ± 6.4	0–15
	Water	8.0 ± 8.2	0–25
	Positive control	14.4 ± 7.7	0–30

Data are mean ± SD of 31 determinations for each plant crude extract or control antibiotic.

**Table 3:** Minimum inhibitory concentrations (MIC_50_) of plant crude extracts and control antibiotics (mg/mL).

Crude extracts/Antibiotics						
*H. pylori* strains	AC	AS_1_	AS_2_	EG	AMPC	MNZ
PE2A	0.312	0.06	0.47	5.0	0.32	1.25
PE5A	0.625	0.12	0.63	1.25	0.63	1.25
PE9C	1.25	0.08	0.63	1.25	0.01	2.5
PE11A	0.156	1.25	0.16	5.0	0.04	5.0
PE11C	0.156	0.08	0.63	1.25	0.04	1.25
PE14C	0.156	0.06	0.47	1.25	0.31	1.25
PE26A	0.312	0.63	1.25	1.25	0.63	1.25
PE70A	0.625	0.06	0.94	1.25	0.32	2.5
PE76A	1.25	2.5	2.5	5.0	0.31	5.0
PE84C	0.625	1.25	–	1.25	0.63	1.25
PE93A	0.08	0.06	0.63	1.25	0.32	2.5
PE93C	1.25	0.16	0.47	1.25	0.63	1.25
PE102C	0.156	0.31	2.5	1.25	0.63	1.25
PE115A	0.312	0.63	1.25	1.25	0.31	5.0
PE155A	2.5	0.08	0.94	1.25	0.16	2.5
PE219C	1.25	0.94	–	1.25	0.31	1.25
PE252C	0.312	1.25	2.5	1.25	0.63	2.5
PE258C	0.312	0.94	2.5	1.25	0.08	3.75
PE369A	0.312	0.63	0.31	1.25	0.31	0.08
PE369C	0.625	0.31	0.31	1.25	0.31	2.5
PE397C	0.312	0.08	1.5	5.0	0.01	5.0
PE402A	1.25	0.06	0.49	5.0	0.32	2.5
PE411A	0.625	0.08	0.63	1.25	0.08	2.5
PE411C	1.25	0.63	0.63	1.25	0.31	1.25
PE430A	0.312	1.25	–	1.25	0.31	1.25
PE430C	0.625	0.31	0.47	1.25	0.31	1.25
PE435A	0.625	0.63	1.25	0.63	0.31	1.25
PE436A	1.25	0.08	0.63	0.63	0.31	0.04
PE436C	0.312	1.25	2.5	1.25	–	1.25
PE466C	0.312	0.36	2.5	1.25	0.31	1.25
NCTC 11638	0.312	0.63	2.5	2.5	0.001	0.004
Mean ± SD	0.63 ± 0.53	0.54 ± 0.56	1.0 ± 0.85	1.85 ± 1.42	0.30 ± 0.20	2.1 ± 1.42

AC, Acetone extract of *C. molle*; AS_1_, Acetone extract of *S. birrea*; AS_2_, Aqueous extract of *S. birrea*; EG, Ethanol extract of *G. kola*; AMPC, Amoxicillin; MNZ, Metronidazole; –, Value not within susceptible range; SD, Standard deviation. The results shown are representative of duplicate determinations for each strain and 31 determinations for each extract or control antibiotic. Strains bearing letter “A” were isolated from the antrum while those bearing letter “C” were isolated from the corpus. The resistant breakpoint for amoxicillin and metronidazole was >0.002 mg/mL and >0.008 mg/mL respectively. Except for the standard control strain, all the strains tested were resistant to amoxicillin and metronidazole giving a percentage resistance of 96.7%.
